# The activity of the intrinsically water-soluble enzyme ADAMTS13 correlates with the membrane state when bound to a phospholipid bilayer

**DOI:** 10.1038/s41598-021-04083-0

**Published:** 2021-12-28

**Authors:** Andrej Kamenac, Tobias Obser, Achim Wixforth, Matthias F. Schneider, Christoph Westerhausen

**Affiliations:** 1 Experimental Physics I, Institute of Physics, University of Augsburg, 86159 Augsburg, Germany; 2 Center for NanoScience (CeNS), Ludwig-Maximilians-Universität München, 80799 Munich, Germany; 3 Department of Dermatology and Venerology, Center for Internal Medicine, University Medical Center Hamburg-Eppendorf, 20246 Hamburg, Germany; 4 Medical and Biological Physics, Technical University Dortmund, 44227 Dortmund, Germany; 5 Physiology, Institute of Theoretical Medicine, University of Augsburg, 86159 Augsburg, Germany

**Keywords:** Biophysics, Biochemistry, Biocatalysis, Biological physics, Phase transitions and critical phenomena

## Abstract

Membrane-associated enzymes have been found to behave differently qualitatively and quantitatively in terms of activity. These findings were highly debated in the 1970s and many general correlations and reaction specific models have been proposed, reviewed, and discarded. However, new biological applications brought up the need for clarification and elucidation. To address literature shortcomings, we chose the intrinsically water-soluble enzyme a disintegrin and metalloproteinase with a thrombospondin type 1 motif, member 13 (ADAMTS13) and large unilamellar vesicles with a relative broad phase transition. We here present activity measurements of ADAMTS13 in the freely dissolved state and the membrane associated state for phosphocholine lipids with different acyl-chain lengths (13:0, 14:0 and 15:0) and thus main phase transition temperatures. While the freely dissolved enzyme shows a simple Arrhenius behavior, the activity of membrane associated ADAMTS13 in addition shows a peak. This peak temperature correlates with the main phase transition temperature of the used lipids. These findings support an alternative theory of catalysis. This theory predicts a correlation of the membrane associated activity and the heat capacity, as both are susceptibilities of the same surface Gibb’s free energy, since the enzyme is attached to the membrane.

## Introduction

Enzymes are crucial to enable and maintain life by accelerating extremely slow chemical reactions. Increasing the temperature typically results in increased reaction rates. The temperature dependence of the reaction rate for freely dissolved enzymes is classically described by an Arrhenius-behavior. Many enzymes in living organisms are not freely dissolved in bulk liquids but bound to surfaces. In fact, the amount of free water in cells makes up only about 20%^1^, so surfaces are of highest importance for *in cellulo* processes and conditions. This holds especially for enyzmes. When they are bound to a lipid membrane, many enzymes change their properties and temperature dependencies^2^. In the 1970s, many studies have found deviations from a pure Arrhenius-behavior. More precisely, they found discontinuities and kinks in the Arrhenius plots of membrane associated enzymes. To mention a few examples, this was observed for (Na^+^-K^+^)-ATPase^3–7^, Ca^2+^-ATPase^8–12^, Cr_55_-isoprenoid alcohol kinase^13–15^, phospholipase^16–19^, acetylcholinesterase^20–22^, and various other enzymes^23–29^.

These kinks in the enzyme activity have been investigated for a correlation with the phase transition temperature of the lipid membrane. However, the provided explanations of reported correlations were individual for each case and partly indistinct. For example, the term ‘viscotropic regulation’ was introduced^4^ in order to explain the correlation of the membrane associated enzyme activity and the phase state of the membrane. The authors of this study concluded an “[...] influence of fatty acyl-chain fluidity on enzyme functions [...]”^4^. This explanation has been reviewed by Sandermann and found to be “[..] rather vague and only loosely defined”^30^.

The studies from Enoch et al.^31^ and Kumamoto et al.^24^ aim further towards a new theory of catalysis. Enoch et al*.* observed the classic discontinuity in the enzyme activity, where they used 14:0 PC membrane and a certain amount of cytochrome b5. When they increased the amount of enzyme, the kink disappeared. They concluded that lateral diffusion became rate limiting. However, when they slightly modified the systems components to test their conclusion, they found that the discontinuity was caused by a change in the catalytic mechanism, as reviewed by Sanderman^30^. This assumption is supported by Kumamoto et al.^24^, who conclude that “[a] theory that employs the phase change can adequately accommodate all the data for temperature [‘]breaks[’] in Arrhenius plots”^24^. Subsequently, they emphasize the need of universality of the theory to be applicable to different membranes and enzymes. The authors state that the theory should be about the general field of catalysis.

Analogous experiments have been published for lipid monolayer associated enzymes. Fichtl investigated the activity of several lipid monolayer associated enzymes concerning their activity^32^. Here, the activity of imbedded horseradish peroxidase under an isothermal expansion of an 1,2-dipalmitoyl-sn-glycero-3-phospho-(1′-rac-glycerol) (DPPG) monolayer was measured. A strikingly similar shape of the enzyme activity and the lipid monolayer compressibility was found. From the thermodynamic point of view, compressibility is—just like the excess heat capacity—a susceptibility. Both are derivatives of the Gibb’s free energy and are mutually coupled^33–35^. This correlation of compressibility and activity is conserved, even for variations of the lipid type (DMPS) and the pH value.

Very similar results have been found for acetylcholinesterase^36^. These results show that not the distinct pH value, lipid or enzyme is crucial for the maximal activity. Rather, the enzyme activity depends on the phase state. This underlines the thermodynamic behavior of membrane associated enzymes and makes a step towards the demanded universal explanation of catalysis. Moreover, universal physical principles finding their way into biochemistry was recently demonstrated in another publication^37^, connecting catalytic rate, Gibbs free energy, and motility.

The conservation of the effect of the phase transition for variation of the enzyme, pH and lipid emphasizes the physical nature and thermodynamic behavior of the membrane associated enzyme activity. This does not only hold for quasi-static systems, but also for dynamical ones, as Schneider and coworkers demonstrated. They applied pH-pulses to DMPS monolayers and measured the activity of embedded acetylcholine esterase to demonstrate the applicability for dynamic changes of the phase state^38^. A propagating pH-pulse can evoke a propagating, local phase transition. When the pulse front is spatially far away from the monolayer associated enzyme, its activity remains constant. Once the pulse reaches the enzyme´s environment, the activity can be enhanced by an order of magnitude. This study is part of a series of publications establishing a new thermodynamic nerve signaling theory^39^, where the action potential is described by an acoustic-like pulse propagating laterally on the membrane. In the context of trigger- and detector potential of phase state sensitive enzymes at the synaptic cleft^40^, it would be of highest interest if the demonstrated phenomena also hold for bilayer associated enzymes.

The underlying theory originally has been published by Kaufmann^41^. Schneider and coworkers picked up and elaborated on certain issues, improved accessibility and applicability^42–44^. In brief, Kaufmann’s theory considers the state of the interface the enzyme forms with its surrounding and applies Einstein’s^45^ ansatz to this interface. To determine the state of the interface, it is crucial to realize, that the enzyme is part of the lipid membrane, i.e. the lipid membrane and the enzyme share one continuous surface of bound water, which is relatively decoupled from the bulk water due to the impedance mismatch^46^. In contrast, the lateral coupling is strong. According to Einstein, the mutual water interface has its own thermodynamic potential^47^. The second derivatives from this thermodynamic potential—the susceptibilities, and thus fluctuations—are coupled^33,34^. This includes fluctuations in enthalpy, area per lipid molecule, charge, and also membrane thickness.

We would like to emphasize that various microscopic models associated to fluctuations, e.g. out of plane membrane fluctuations^48^ might exist, contributing to explain the total increased activity. However, all of them are included in this statistical approach of an entropy potential of the interface, and thus a special case or part of the overall phenomenon.

These susceptibilities of such a system are easily measurable, e.g., heat capacity via calorimetry, or compressibility via Langmuir trough. Moreover, heat capacity and fluctuations are directly related by the fluctuation theorem^42^:

$$c_{p} = \frac{{{\text{d}}\left\langle H \right\rangle }}{{{\text{d}}T}} = \frac{{\left\langle {H^{2} } \right\rangle - \left\langle H \right\rangle^{2} }}{{RT^{2} }}.$$

If one susceptibility exhibits a peak, e.g., the lipid membrane undergoes a melting transition, due to the coupling, all susceptibilities will likely exhibit a peak. As the enzyme is part of the surface and shares a mutual thermodynamic potential, the enzyme activity is another susceptibility and is predicted to display a peak as well^32,43,46^.

For the study presented here, we designed an experiment to further elucidate the correlation of the membrane associated enzyme activity and the excess heat capacity along the same lines as summarized above. Therefore, we chose particularly a water-soluble enzyme, which is by its nature not associated in any way to a lipid membrane. This lack of evolutionary bias makes this enzyme an excellent choice to investigate the correlation of membrane phase transitions and enzyme activity.

In this article, we quantify the activity of ADAMTS13 in absence and presence of lipid vesicles as a function of temperature. In this temperature range, the lipid vesicles are at different phase states, which we quantified by calorimetric measurements. Additionally, we vary the phase state isothermally by using 13:0 PC, 14:0 PC, and 15:0 PC. For this, we measure the activity of ADAMTS13 in parallel at *T* = 24 °C, where 13:0 PC is in the L_α_ phase, 15:0 PC in the L_β’_ phase, and 14:0 PC in the coexistence state. Finally, we discuss the observed correlation in the context of the alternative theory of catalysis.

## Results and discussion

**Figure 1:**
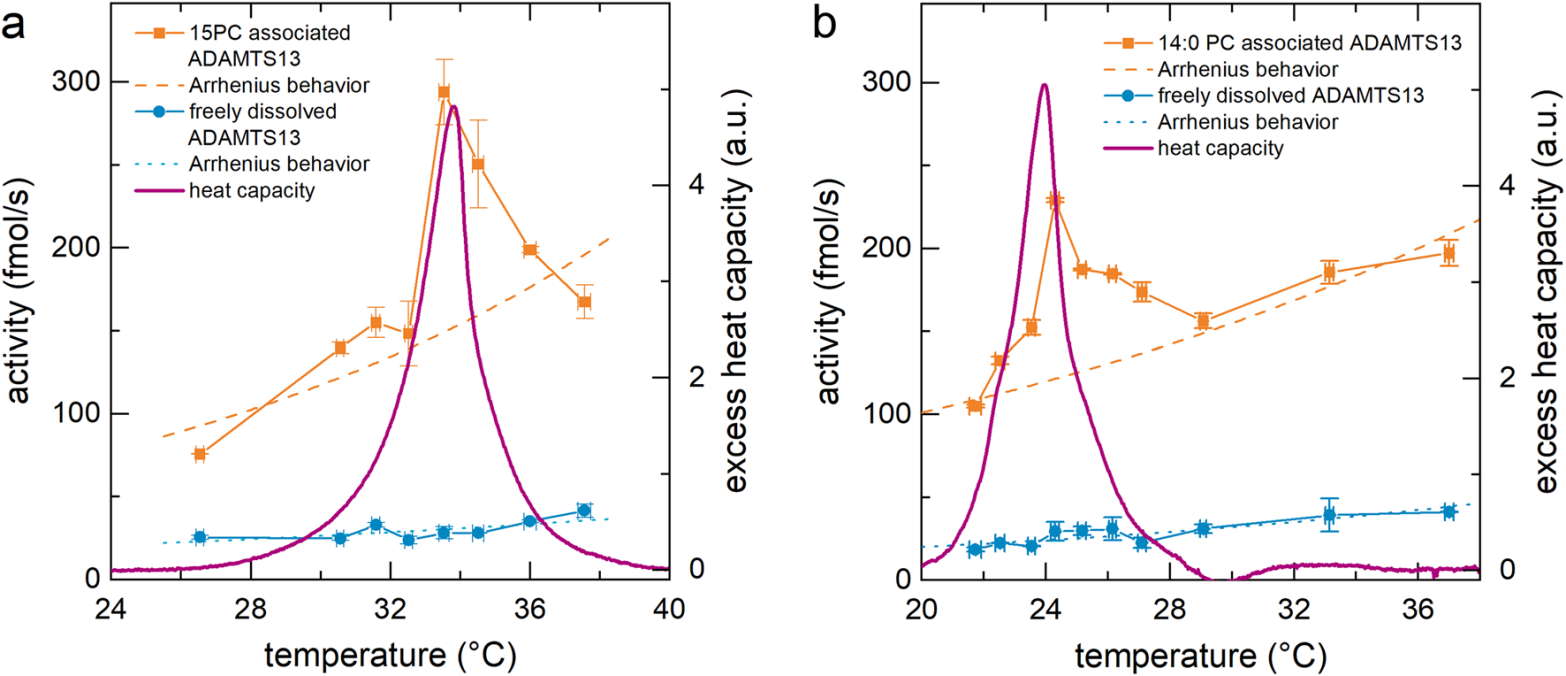
ADAMTS13 activity as a function of temperature and membrane phase state for (**a**) 15:0PC LUV and (**b**) 14:0PC LUV. Blue circles are the reference values for ADAMTS13 in the dissolved state. Orange Squares represent the activity for ADAMTS13 at 15:0 PC and 14:0 PC membranes, respectively. The pink solid line represents the excess heat capacity of the respective system. The activity of membrane associated enzyme was corrected according to the “Supplementary Information” by a simple factor due to optical reasons. Error bars represent the standard deviation of three independent measurements.

Figure 1a shows the excess heat capacity of the 15:0 PC containing sample (solid line). The main phase transition temperature is in accordance with literature^49^. The width of these LUV excess heat capacity curves is higher compared to MLV samples. This can be understood as a consequence of reduced cooperativity^42^. Moreover, Fig. 1a shows the steady state activity of ADAMTS13 as a function of temperature in a freely dissolved state (blue circles) and associated to 15:0 PC membranes (orange squares). The activity of the freely dissolved enzyme increases with increasing temperature (blue circles) in accordance with an Arrhenius behavior, as reported earlier in literature^50^. In contrast, ADAMTS13 bound to 15:0 PC membranes shows an overall higher activity and a pronounced maximum at *T* = 34 °C. This activity maximum temperature matches the excess heat capacity maximum. We would like to emphasize, that this is an inherent qualitative difference to the dissolved state. In particular, between 33 °C ≤ *T* ≤ 38 °C, the activity decreases with increasing temperature.

It is intriguing, that the membrane associated ADAMTS13 activity is overall elevated in comparison to its activity in solution. At the peak activity, the membrane associated ADAMTS13 is increased by a factor of about 10 compared to the reference.

To exclude an eventual evolutionary bias in terms of temperature optimization—which literature does not observe^50^—or any kind of system specific coincidence, we exchanged the membrane lipids from 15:0 PC to 14:0 PC. 14:0 PC leaves the chemical situation identical, as the head groups are identical. The one acyl-unit shorter chain length slightly decreases the thickness of the membrane, from which no interactions with the enzyme are expected, as ADAMTS13 is bound peripherically. Most importantly, the lipid exchange from 15:0 to 14:0 PC substantially shifts the phase transition temperature of the system from *T* = 33 °C to *T* = 24 °C. As shown in Fig. 1b, this shift in excess heat capacity to *T* = 24 °C is accompanied by a shift in the activity peak to *T* = 24 °C, as well. Again, this peak activity temperature matches with the maximum excess heat capacity temperature, analogous to Fig. 1a. The peaks are similar, as the FWHM is identical for both excess heat capacities and for both activities respectively, as shown in the Supplementary Information Fig. S3. We would like to emphasize, that this does not result in a simple kink in the Arrhenius plot, as reported in some studies on other enzymes in the presence of membranes, as summarized in the introduction section.

**Figure 2:**
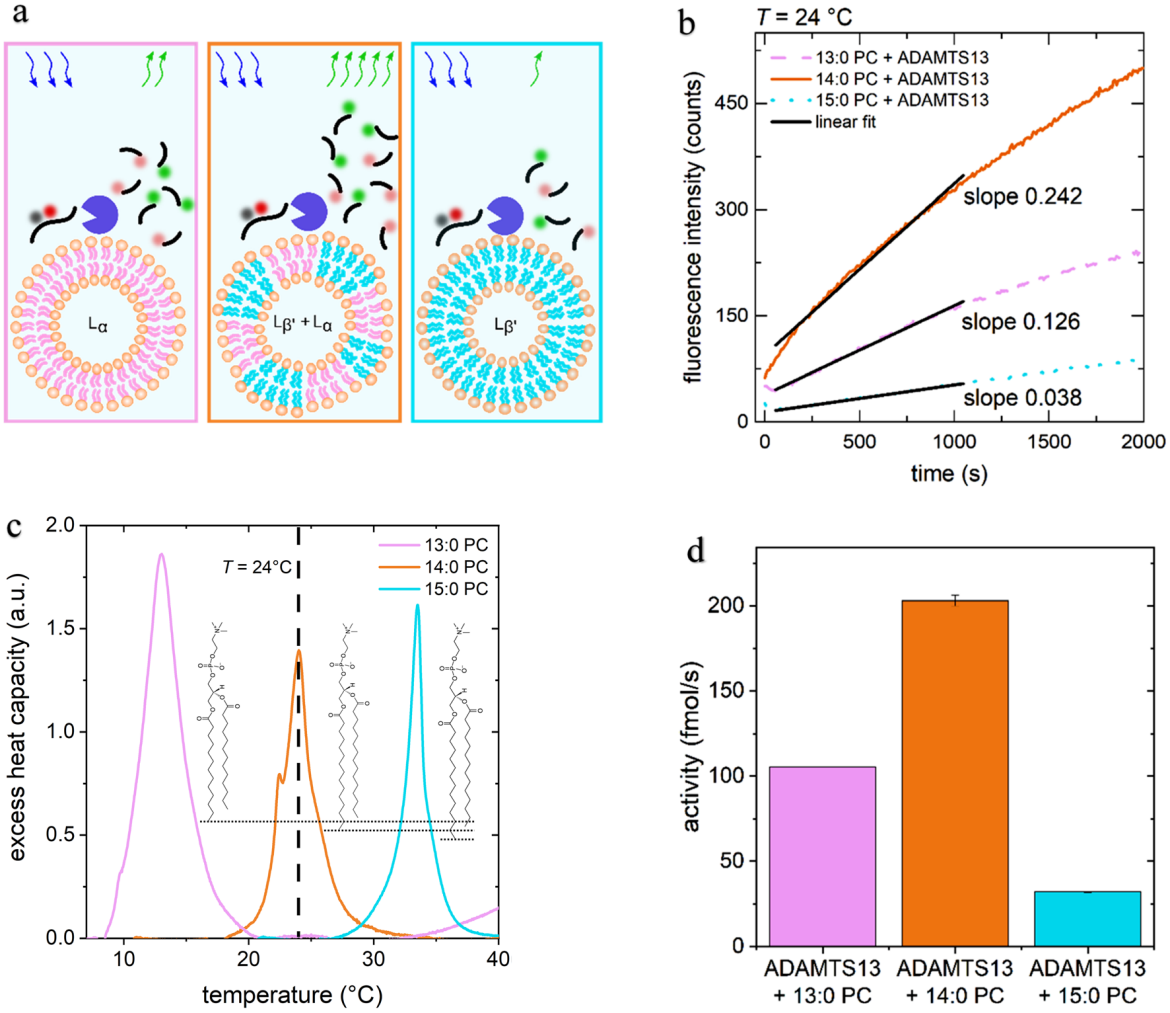
(**a**) Illustration of the experimental setup: ADAMTS13 (purple circle segment) was attached to three different liposomes: 13:0 PC (left), 15:0 PC (right), and 14:0 PC (middle), FRET-substrate was added (black with colored dots). At 24 °C, the phase states of the liposomes are respectively L_α_, L_β’_, and in coexistence, respectively, as illustrated by their acyl-chain shape and color. (**b**) ADAMTS13 kinetics for the 13:0 PC, 14:0 PC, and 15:0 PC samples at T = 24 °C. The enzyme bound to 14:0 PC clearly displays the highest activity. (**c**) Excess heat capacity of LUV of three phosphocholines with different acyl-chain lengths. At 24 °C all three membranes are in a different phase state: 13:0 PC is L_α_, 15:0 PC is L_β’_, and 14:0 PC is in the coexistence regime. (**d**) ADAMTS13 activity determined from the kinetics shown in (**b**).

In principle, the finding of a pronounced activity peak, as shown in Fig. 1, on its own shows the dependence on the thermodynamic state of the membrane-enzyme-system. However, to improve comparability and exclude any eventual artifacts arising from the presence of a soft interface, like lateral diffusion of substrate along the membrane, we conducted an additional experiment. In this experiment, we conducted activity measurements with 13:0 PC, 15:0 PC, and 14:0 PC LUV isothermally (*T* = 24 °C). The kinetics were measured simultaneously, which eliminated any doubts related to enzyme aging or day-to-day experimental ambiguities. As illustrated in Fig. 2a and as quantified in Fig. 2c, at this temperature, all three phase states are represented: 13:0 PC being far in the L_α_ state, 15:0 PC being far in the L_β′_ state, and 14:0 PC being at its main phase transition.

Figure 2b,d clearly show the highest activity for 14:0 PC. The enzyme bound to the L_α_ phase membrane (13:0 PC) exhibits roughly half the activity compared to 14:0 PC, and the enzyme bound to the L_β’_ state membrane (15:0 PC) exhibits an activity of almost an order of magnitude less. This is in qualitative agreement with Fig. 1 and convincingly demonstrates the correlation of the enzyme activity with the heat capacity of the lipid membranes. In addition, this supports the conclusion that membrane fluidity is not the crucial parameter here, as the 13:0 PC samples at *T* = 24 °C have a higher fluidity than the 14:0 PC samples, but show only about half the activity.

**Figure 3:**
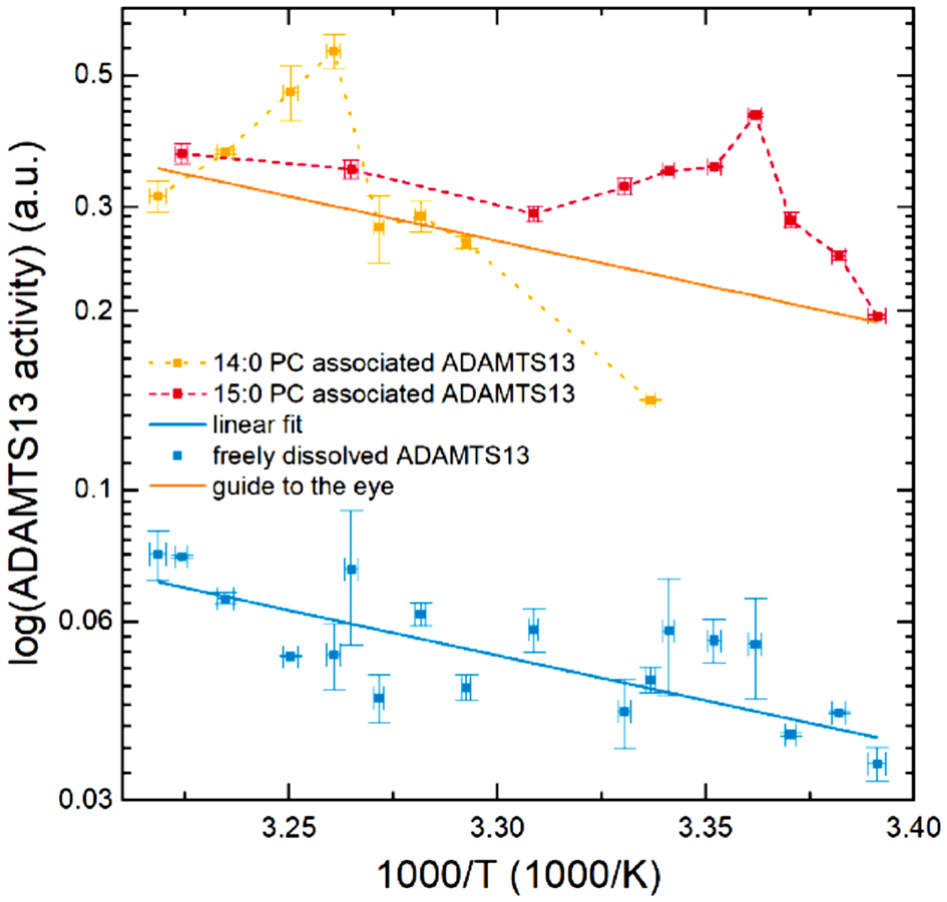
Arrhenius plots of the data shown in Fig. 1. The plot does not show kinks or discontinuities, but peaks at the phase transition temperature for the membrane associated enzyme.

Figure 3 shows Arrhenius plots to allow for comparison with studies cited in the introduction. This shape of an Arrhenius plot is entirely different from the according one of dissolved ADAMTS13^50^, as both plots of the membrane associated samples show a pronounced peak. Interestingly, this shape has been reported before by Lehto and Sharom^19^ for the activity of Phospholipase C at DMPC membranes, but the interpretation was vastly different. They interpreted it in the context of biphasic curves as an analogue triphasic curve and therefore equivalent to previous literature. It was constituted for the non-Arrhenius behavior to arise from a changing collision frequency with increasing temperature due to Δ*S* at the phase transition.

Another interpretation, which was popular later in the 1970s, was the assumption for these findings to be an effect caused by phase separation. According to this theory, the phase separation would occur at the transition temperature and would act as a solubilizing agent for the substrate or enzyme. In the latter case, “[T]he motional state of the lipid phase will then determine the rotational and lateral diffusion rates of the solubilized protein, and probably its ability to undergo conformational changes”^30^. Furthermore, in general enhanced transport of substrate to a membrane bound enzyme at the phase transition within or along the membrane might very well play a role. However, we do not expect this to have any effect on the activity, as the enzyme is very slow and we work in substrate saturation, at least for the analyzed first minutes of the kinetic. To further unravel the origin of the non-linear temperature dependence of the enzyme activity, one could take into consideration that the membrane-bound enzymes effectively do not work in substrate saturation. But, as has been shown for the vesicle-free samples, we obtain a perfectly linear dependence of the determined activity and the enzyme concentration used. Thus, for vesicle free samples, substrate saturation can be safely assumed. Second, in general, we obtained higher activities for membrane bound enzyme compared to freely dissolved enzyme. If we had a non-saturated situation for the membrane-bound enzymes, the activity should be decreased, not increased. Moreover, this effect had to be non-linearly temperature dependent. However, reference experiments with freely dissolved enzyme in samples with and without vesicles show identical activities (Fig. S5). This indicates that no significant substrate-membrane binding influences the enzyme activity.

Kimelberg et al.^3^ report varying positions of the kinks with respect to the phase transition temperature. They find the kinks to occur both at the beginning of the main phase transition and at the end of the transition range, for various lipids. For others, the kink positions vary further. However, they argue that slight variations of the aqueous environment, like *c* = 3 mM Mg^2+^, shift the heat capacity profile of DPPG liposomes by Δ*T* = 10 K. This is also true for proteins, although they measured pure phospholipid vesicles calorimetrically, instead of measuring the whole system for activity-transition correlation purposes. In addition, the dynamic enzyme regulation was proposed: “It is quite possible that changes in membrane lipid fluidity could be involved both in physiological controls as well as pathological situations”^4^.

An alternative and general explanation for the observed striking correlation of phase transition of the according lipid membrane and enzyme activity is provided by Kaufmann’s theory^41,43^ as introduced above. This theory is perfectly in line with these and other experimental findings, which observe peaks correlating with the phase transition temperature. It is an exciting question to study, how far also phase transitions in living cells, which exist, are relevant for the regulation of catalytic activity^51^. There exist interesting reports on the correlation of ATPase activity with the onset of phase transitions in biological membranes, while this is not the case for NADH-oxidase in the same membrane^52^. This could be interpreted in the context of different local environments within the heterogeneous membrane resulting in a variation of phase transition temperatures.

Furthermore, for experiments, which have not observed peaks but kinks or discontinuities, the theory predicts peaks at the intersection region of the kinks and discontinuities. Potential reasons why the authors of these publications did not observe such maxima, could be amongst others broad finite temperature steps of the kinetic method combined with the sharpness and sensibility of the phase transitions to environmental conditions as contamination or (microenvironmental) pH-shifts. These pH-changes could be a consequence of high enzyme activity leading to protonation. In turn, protonation especially of charged lipids, can cause drastic changes of the membrane state and therefore theoretically self-regulation of the enzyme activity. A reason why this does not happen here could be the comparably low turnover number of ADAMTS13. Moreover, Fichtl et al. already showed for lipid monolayers, that local acidification can create pulses that propagate along the interface with the velocity of sound and that these propagating local variations of the membrane state could regulate enzyme activity at elsewhere on the cell surface^32,38^.

## Conclusion

The correlation of the membrane associated enzyme activity and the lipid membrane phase transition for different lipids has been shown. ADAMTS13, being a water-soluble enzyme, makes this correlation more persistent, as its native environment is in the blood circulation, not lipid membranes. Artifacts, like lateral diffusion as an activity enhancement could be excluded theoretically and due to substrate abundance. An activity enhancement due to increased fluidity, as it is proposed by Kimelberg et al.^3^ could be experimentally excluded as well. Summarized, the classical induced fit theory cannot fully explain these experimental findings as well as those from several other mentioned publications^38,53–58^. A broader, more general theory of catalysis is needed to properly treat the presented literature and experimental findings. Such a general theory of catalysis has been provided by Kaufmann and specified by Schneider and coworkers as introduced above.

This new theory of catalysis takes a thermodynamic approach and provides a simple explanation for the effects observed here without the need of a molecular picture. At phase transitions, the entropic potential of the enzyme-lipid-system broadens. Thus, fluctuations increase sharply, also in the substrate-enzyme complex, fostering the catalytic activity of the enzyme.

Our data shown here, in addition to the extensive studies on enzymes at lipid monolayers, further support the idea and might contribute to the discussion how this sensitivity of enzyme activity towards the membrane state can contribute to communication on the cellular level^38,40^.

## Experimental

### Vesicle extrusion

LUV were produced by employing extrusion^59^. Briefly, 13:0 PC, 14:0 PC or 15:0 PC and 1,2-dioleoyl-sn-glycero-3-[(*N*-(5-amino-1-carboxypentyl)iminodiacetic acid)succinyl] (nickel salt) (18:1 DGS-NTA (Ni)) were mixed at a molar ratio of 98:2 in a glass vial. To obtain a lipid cake, a gentle N2 stream evaporated most of the chloroform, followed by a complete extraction under vacuum overnight. The lipid cake was then hydrated using the assay buffer (ACTIFLUOR™ ADAMTS13 Activity Assay, LOXO GmbH, Germany), and sonicated at Δ*T* = 20 K above the lipid phase transition temperature for *t*_S_ = 30 min, followed by another *t*_h_ = 30 min healing time to obtain multilamellar vesicles. The multilamellar vesicle solution was then loaded into the Hamilton syringes and kept Δ*T* = 20 K above their respective phase transition temperature. Meanwhile, the extruder was assembled where *d* = 0.1 µm diameter polycarbonate membranes (Whatman, USA) were used. Once temperature has equilibrated on all parts of the extruder (Avanti Polar Lipids, USA), the multilamellar vesicle solution is pushed back and forth at least ten times through the polycarbonate membrane. In the end, at the final lipid concentration *c*_L_ = 3 mM, the LUV solution is opalescent to the naked eye, whereas the MLV solution is milky. All extruder components were purchased from Avanti Polar Lipids. The lipids were purchased from Avanti Polar Lipids dissolved in chloroform.

### Differential scanning calorimetry

All liquids were degassed prior to the calorimetry (MicroCal VP-DSC, MicroCal Inc., now Malvern Panalytical Ltd, UK) at *p* = 15 psi pressure. A scan rate *β* = 15 K/h was chosen to ensure a quasi-static process. From three subsequent up and down cycles, the last up scan was evaluated. The baseline was determined and subtracted as described previously^42^. For the reference, we used assay buffer solution. For the sample, we used the same concentrations as used in the activity measurement, including vesicles, enzyme, substrate, and buffer.

### Enzyme activity assay

To measure the ADAMTS13 activity, we used a commercially available fluorometric assay (ACTIFLUOR™ ADAMTS13 Activity Assay, LOXO GmbH, Germany, based on the study by Kokame et al.^60^). The buffer provided in the assay kit was used. The enzyme provided by the assay kit was only used for complementary measurements in the “Supplementary Information”. All measurements presented in the figures were conducted with his-tagged ADAMTS13 to enable enzyme binding to the membrane. This enzyme was synthesized as follows. In the previously described ADAMTS13-expression vector was inserted a c-terminal 6xHis-Tag by in vitro mutagenesis using the QuikChange site directed mutagenesis kit (Stratagene)^61^. The transfection and expression was done as previously described^62^. The substrate was prepared and aliquoted according to the assay protocol. Briefly, it was dissolved in DMSO, aliquoted stored in the freezer. Shortly before usage, it was thawed and diluted 25-fold using assay buffer. ADAMTS13-his stock solution was diluted 4-fold using assay buffer.

### Experimental procedure

The setup is illustrated in Fig. 4. Continuous fluorometric kinetics were performed using a plate reader (Tecan Infinite^®^ F200 PRO, excitation at $\lambda_{ex}$ = 485 nm, emission at $\lambda_{em}$ = 535 nm) and 96-well plates (Greiner, flat, black, Cat. No. 237105). The plate reader temperature control was adjusted and equilibrated at least *t* = 15 min before insertion of the well plate and another *t* = 15 min before starting the reaction.

For the experimental design, doublets of freely dissolved ADAMTS13 (approx. *c*_ADAMTS13_ = 1 nM) and membrane associated ADAMTS13 and enzyme free references were carried out simultaneously at constant temperature. The substrate concentration is approximately *c*_Substrate_ = 7.2 µM.

### Activity evaluation (Fig. 4)

**Figure 4:**
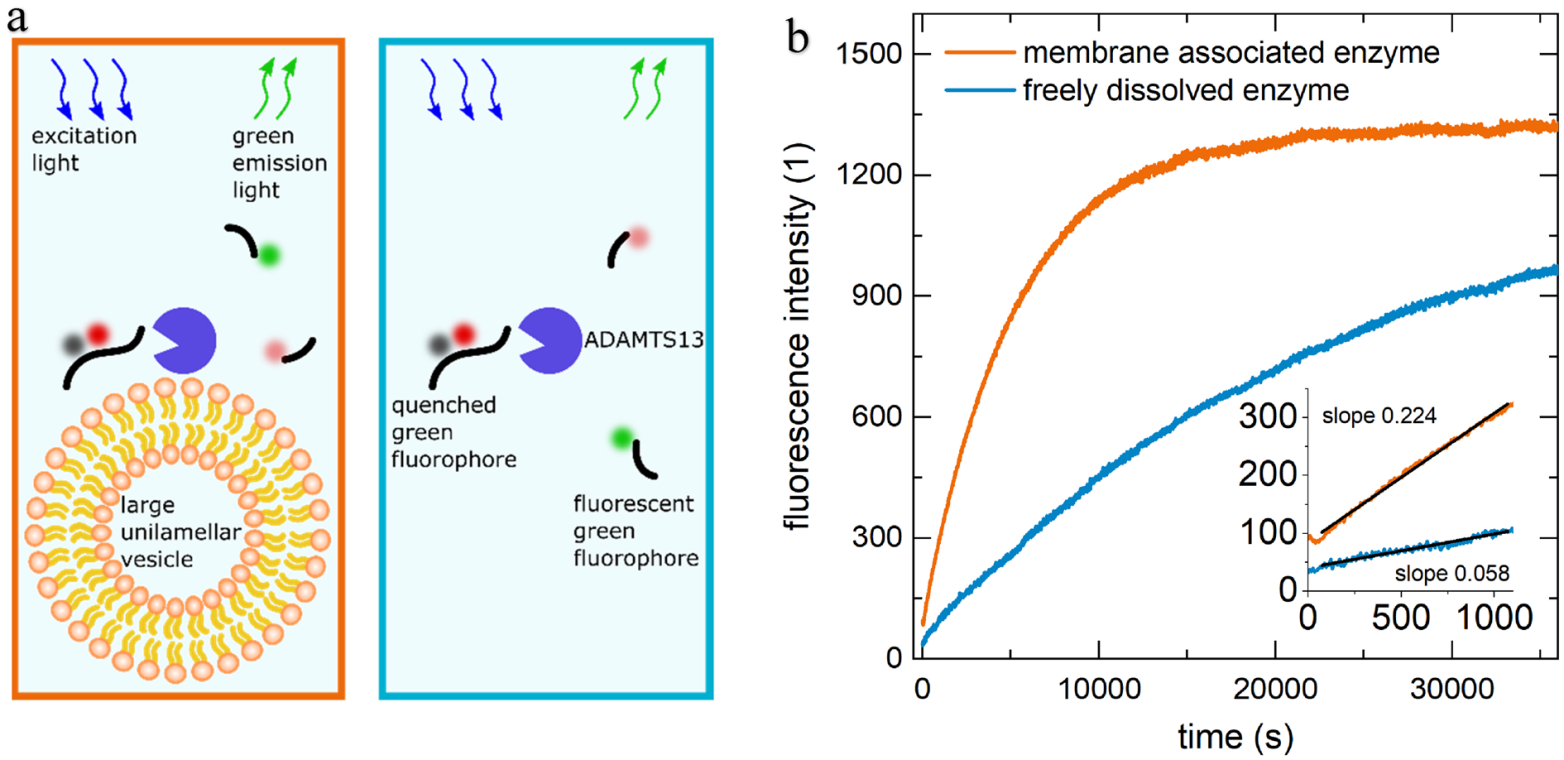
(**a**) Illustration of the experimental design. One well (left, orange frame) contains the substrate, enzyme, and vesicles, whereas in the other (right, blue frame), the vesicles are substituted by buffer. The assay is based on fluorescence-resonance-energy-transfer (FRET). Embedded ALEXA fluorochromes in the A2 domain of VWF (Von-Willebrand-Factor) are decoupled after cleaving of the substrate, this leads to an increase of the green fluorescence. (**b**) Typical kinetic measurement (T = 24 °C) of freely dissolved enzyme (blue) and membrane (14:0 PC) associated enzyme (orange).

## Supplementary Information

Supplementary Figures.

## Abbreviations

ADAMTS13: A disintegrin and metalloproteinase with a thrombospondin type 1 motif, member 13
14:0 PC: 1,2-Dimyristoyl-sn-glycero-3-phosphocholine
DMPC: 1,2-Dimyristoyl-sn-glycero-3-phosphocholine
15:0 PC: 1,2-Dipentadecanoyl-sn-glycero-3-phosphocholine
LUV: Large unilamellar vesicles
DPPG: 1,2-Dipalmitoyl-sn-glycero-3-phospho-(1′-rac-glycerol)
13:0 PC: 1,2-Ditridecanoyl-sn-glycero-3-phosphocholine
