# Supplementary material for: The activity of the intrinsically water-soluble enzyme ADAMTS13 correlates with the membrane state when bound to a phospholipid bilayer
Source: Sci Rep. 2021 Dec 28;11:24476. doi: 10.1038/s41598-021-04083-0 (PMC8714821; doi:10.1038/s41598-021-04083-0)
Supplement: Supplementary file 1 — Supplementary Figures. [file 41598_2021_4083_MOESM1_ESM.pdf]

Supplementary Information to

The activity of the intrinsically water-soluble enzyme ADAMTS13 correlates with the membrane state when bound to a phospholipid bilayer

Andrej Kamenac[1,2], Tobias Obser[3], Achim Wixforth[1,2], Matthias F. Schneider[4], Christoph Westerhausen[1,2,5,*]

[1] Experimental Physics I, Institute of Physics, University of Augsburg, 86159 Augsburg, Germany
[2] Center for NanoScience (CeNS), Ludwig-Maximilians-Universität München, 80799 Munich, Germany
[3] Department of Dermatology and Venerology, Center for Internal Medicine, University Medical Center Hamburg-Eppendorf, 20246 Hamburg, Germany
[4] Medical and Biological Physics, Technical University Dortmund, 44227, Dortmund, Germany
[5] Physiology, Institute of Theoretical Medicine, University of Augsburg, 86159 Augsburg, Germany
* christoph.westerhausen@gmail.com

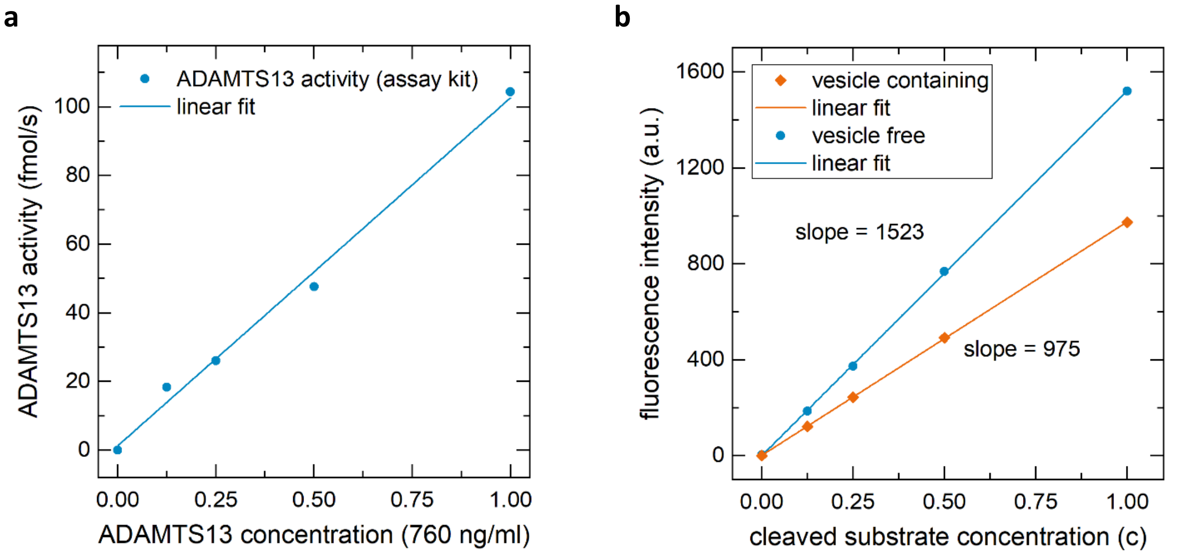

Figure S1 Calibrations. *a) The maximum activity of ADAMTS13 is linearly dependent on the enzyme concentration. This allows a quantitative evaluation of the activity measurements in temperature dependence. According to literature* [63]*, the activity under similar conditions was determined to* k_{cat} *= 4.4* s^{-1} *as an estimate. The estimated activity is 43 fmol/s at 760 ng/ml and 37 °C. b) Fluorescence dampening by LUV and the calibration factor. Cleaved substrate was diluted in pure assay buffer and LUV containing buffer at the same concentration used in Fig. 2. As a result, as the same amount of cleaved substrate shows lower fluorescence intensity in presence of LUV, a correction factor was applied on the values in Fig. 1 and consequently in Fig. 3. The original activity of the vesicle containing specimen was multiplied by the factor 1523/975.*

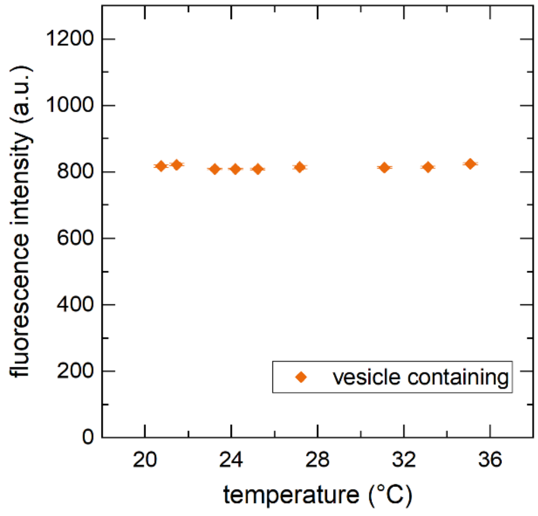

Figure S2: Independence of the fluorescence intensity for constant amount of cleaved substrate from the phase state of the lipid membrane. *The lipid membrane - in this case 14:0 PC - undergoes a phase transition at 24°C, though the fluorescence intensity remains constant over the whole temperature range.*

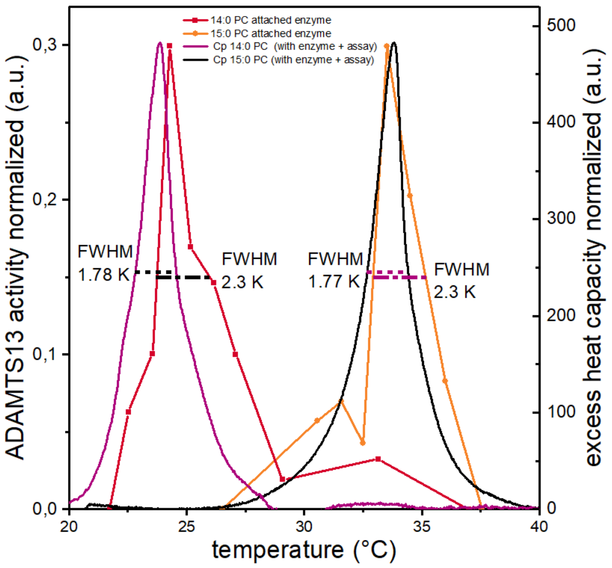

Figure S3: Heat capacity and activity peaks. *The activities were subtracted by a linear baseline and normalized; the excess heat capacity peaks were subtracted by a cubic baseline. Both excess heat capacity peaks display an identical FWHM of 1.78 K. The activity peak-shape was not fitted but simplified as the line-symbol plot and display also an identical FWHM of 2.3 K, which is about 30% higher than the FWHM of the excess heat capacity.*

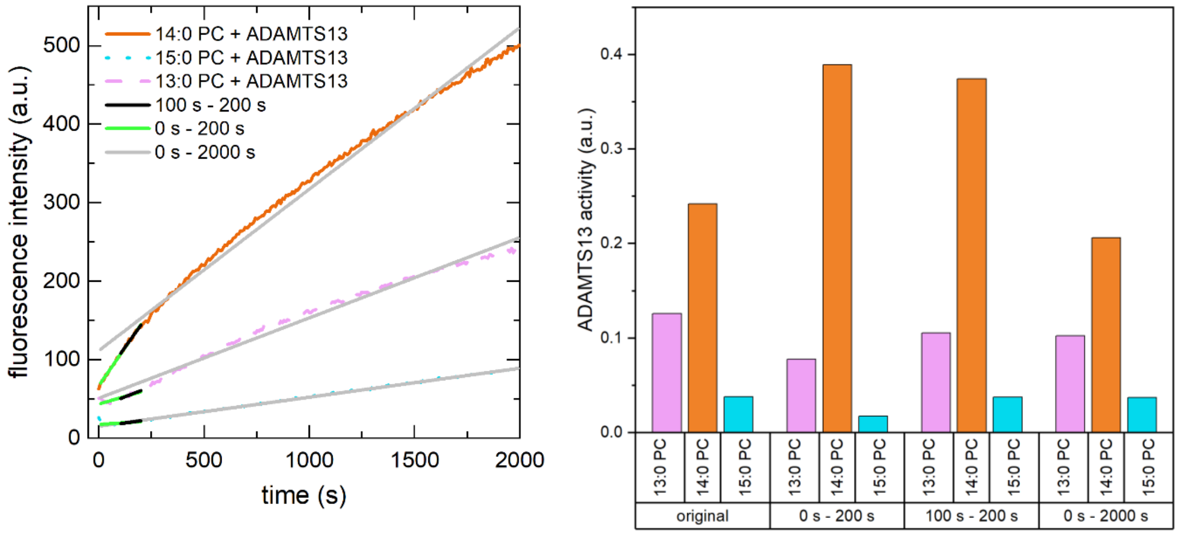

Figure S4: Influence of the kinetic fitting range. *a) ADAMTS13 kinetics for the 13:0 PC, 14:0 PC, and 15:0 PC samples at T = 24 °C shown in Fig. 2b in the manuscript fitted for different time intervals green: 0s-200s, black 100s-200s, grey: 0s-2000s. b) dependence of the obtained activities from the fitting interval in a) compared with the original fitting interval presented in the manuscript 50s – 1050 s*

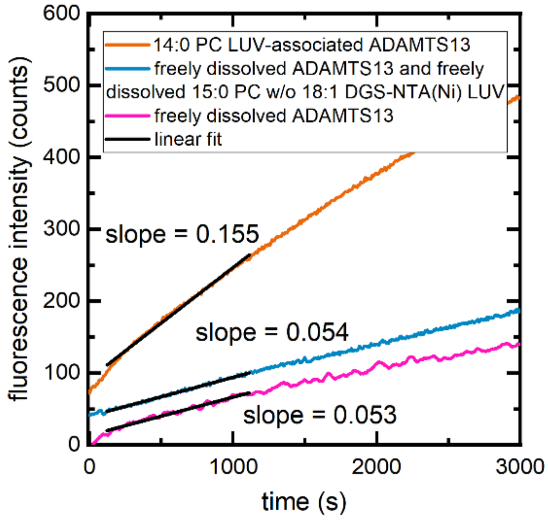

Figure S5: Influence of the presence of lipid membranes for freely dissolved enzymes *ADAMTS13 kinetics at T = 24 °C for 14:0 PC samples containing 18:1 DGS-NTA(Ni) to bind the enzyme via its his-tag (orange), 15:0 PC samples containing no 18:1 DGS-NTA(Ni) resulting in freely dissolved enzyme (blue) and freely dissolved enzyme without lipid vesicles present (pink). The almost identical activity of the latter two samples indicates, that the pure presence of a lipid membrane does not influence the enzyme activity by binding a significant amount of the substrate.*
